# Cumulative Live Birth Rate and Cost-Effectiveness Analysis of Gonadotropin Releasing Hormone-Antagonist Protocol and Multiple Minimal Ovarian Stimulation in Poor Responders

**DOI:** 10.3389/fendo.2020.605939

**Published:** 2021-01-14

**Authors:** Yuan Liu, Rongjia Su, Yu Wu

**Affiliations:** ^1^ Reproductive Medicine Center, Department of Obstetrics and Gynecology, Shanghai General Hospital, Shanghai Jiaotong University School of Medicine, Shanghai, China; ^2^ Department of Obstetrics and Gynecology, Shanghai General Hospital, Shanghai Jiaotong University School of Medicine, Shanghai, China

**Keywords:** *in vitro* fertilization, poor ovarian responders, gonadotropin releasing hormone-antagonist, minimal ovarian stimulation, cumulative live birth rate

## Abstract

**Background:**

The overall cumulative live birth rate (CLBR) of poor ovarian responders (POR) is extremely low. Minimal ovarian stimulation (MOS) provides a relatively realistic solution for ovarian stimulation in POR. Our study aimed to investigate whether multiple MOS strategies resulted in higher CLBR compared to conventional gonadotropin releasing hormone (GnRH) antagonists in POR.

**Methods:**

This retrospective study included 699 patients (1,058 cycles) from one center, who fulfilled the Bologna criteria between 2010 and 2018. Overall, 325 women (325 cycles) were treated with one-time conventional GnRH antagonist ovarian stimulation (GnRH-antagonist). Another 374 patients (733 cycles) were treated with multiple MOS including natural cycles. CLBR and time-and-cost-benefit analyses were compared between these two groups of women.

**Results:**

GnRH antagonists provided more retrieved oocytes, meiosis II oocytes, fertilized oocytes, and more viable embryos compared to both the first MOS (p < 0.001) and the cumulative corresponding numbers in multiple MOSs (p < 0.001). For the first *in vitro* fertilization (IVF) cycle, GnRH antagonists resulted in higher CLBR than MOS [12.92 *versus* 4.54%, adjusted OR (odds ratio) 2.606; 95% CI (confidence interval) 1.386, 4.899, p = 0.003]. The one-time GnRH-antagonist induced comparable CLBR (12.92 *versus* 7.92%, adjusted OR 1.702; 95% CI 0.971, 2.982, p = 0.063), but a shorter time to live birth [9 (8, 10.75) months *versus* 11 (9, 14) months, p = 0.014] and similar financial expenditure compared to repeated MOS [20,838 (17,953, 23,422) ¥ *versus* 21,261.5 (15,892.5, 35,140.25) ¥, p = 0.13].

**Conclusion:**

Both minimal ovarian stimulation (MOS) and GnRH-antagonists provide low chances of live birth in poor responders. The GnRH antagonist protocol is considered a suitable choice for PORs with comparable CLBR, shorter times to live birth, and similar financial expenditure compared to repeated MOS.

## Introduction

Approximately 20% of all women undergoing assisted reproductive technology (ART) treatment demonstrate a poor ovarian response with very few retrieved oocytes, which are of low-quality. Most of these patients have poor ovarian reserve (1). In studies, some poor responders were retrospectively identified after some form of conventional ovarian stimulation. Patients with advanced age or abnormal ovarian reserve tests are more appropriately defined as expected poor responders. The Bologna criteria have been validated to represent a homogenous population with a uniform poor prognosis and similar clinical outcomes. According to the Bologna criteria of the European Society of Human Reproduction and Embryology (ESHRE) consensus, “poor response” to ovarian stimulation for *in vitro* fertilization (IVF) is defined by the presence of at least two of the three following features: 1) age ≥ 40 years or any other risk factor for POR, 2) ≤3 oocytes retrieved previously after conventional stimulation, and 3) antral follicle count (AFC) < 5–7 follicles or anti Mullerian hormone (AMH) < 0.5ng/ml (2).

These patients represent a conundrum in modern IVF. Studies on ART did not provide solid evidence for the preferred strategy and definite solutions for parenthood in these patients, considering the limited supply of oocytes, poor quality of embryos, and high frequency of canceled cycles. However, adjuvant treatments such as growth hormone (GH), dihydroepiandrosterone (DHEA), and CoQ10 have been claimed to be co-treatments of choice for controlled ovarian stimulation (COS) in these patients, and have shown somewhat better clinical results in some studies in terms of achieving pregnancy (3–5). However, the overall pregnancy rate per cycle in PORs is still extremely low, and varies from 7.6 to 17.5% compared to 25.9–36.7% in normal responders (6). The drop-out rate in this population of women is as high as 25% worldwide. In practice, the low live birth rate varies between different POSEIDON groups; this is mainly attributed to maternal age and ovarian response. It is of utmost importance to provide effective and patient-friendly alternative treatment options for poor responders based on the couple’s genetic material.

Several ovarian stimulation protocols have been investigated, including either gonadotropin-releasing hormone (GnRH) agonists or antagonists; however, no consistent results have been reported (7–10). Recently, the DuoStim strategy, which involves luteal-phase stimulation (LPS) and follicular-phase stimulation (FPS) in one single cycle, has been reported to be promising in that it avoids discontinuation after failed attempts and slightly increases the cumulative live birth rate (CLBR) per intention to treat (11). However, cost-benefit analysis and more randomized controlled trials are needed to verify the effectiveness and safety issues. Previous data have demonstrated that increased starting doses in predicted poor responders to IVF/intracytoplasmic sperm injection (ICSI) did not increase the live birth rate, but was more highly priced (12, 13). Although studies on minimal ovarian stimulation (MOS) or modified nature cycles in POR are limited, they have suggested that MOS is a relatively realistic solution for parenthood in POR compared to conventional high dose stimulation. MOS showed a relatively higher implantation rate, acceptable live birth rate, and preferred cost-effectiveness, although fewer oocytes were retrieved (14–20). However, no study has evaluated the CLBR per person for multiple modified nature cycles. CLBR has been a better indicator of quality and success of IVF overall, as multiple cycles of MOS are usually performed instead of one-time stimulation; in addition, cryopreservation has become an integral aspect of IVF (21). It remains unclear whether poor responders could actually benefit from MOS. No data comparing the CLBR between multiple MOS and high-dose GnRH antagonist protocols in POR are available, and studies comparing time and cost effectiveness analysis are lacking. The aim of this study was to evaluate the CLBR and time-and-cost-benefit difference between GnRH-antagonists and multiple MOS protocols in poor responders who fulfilled the Bologna criteria. This study will help clinicians personalize and select a relatively superior COS strategy for these difficult patients.

## Materials and Methods

### Participants

**Figure 1:**
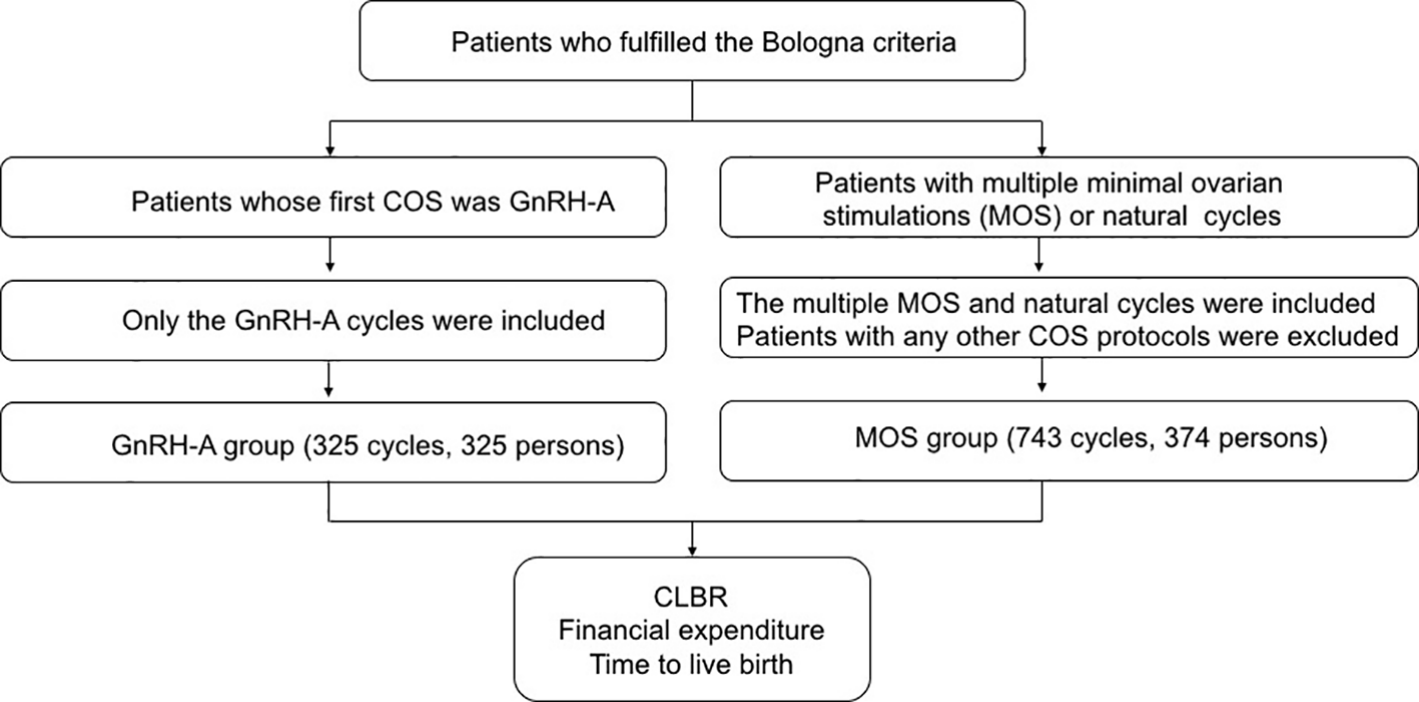
Flow diagram of patient inclusion.

This retrospective study analyzed 325 poor responders who underwent 325 GnRH-antagonist cycles, and 374 poor responders who underwent 733 minimal ovarian stimulation cycles between January 2010 and June 2018 in one assisted reproduction center. Patient inclusion criteria were patients who fulfilled the Bologna criteria for the definition of POR which is defined by the presence of at least two of the three following features: 1) age ≥ 40 years or any other risk factor for POR, 2) ≤3 oocytes retrieved previously after conventional stimulation, and 3) antral follicle count (AFC) < 5–7 follicles or anti-Mullerian hormone (AMH) < 0.5ng/ml. The AFC was determined by counting follicle sized between 2 and 10 mm according to criteria proposed by Broekmans et al. in 2010 (22). AFC observers were trained by arranging workshop and instructions for the procedure. AMH was not included in our analysis due to the inconsistency of detection method in the hospital. Among the patients who fulfilled the Bologna criteria, our analysis included two groups of POR. The first group is poor responders in whom the first stimulation cycle was administered with the GnRH-antagonist protocol. Notably, only the first stimulation cycle namely the GnRH-antagonist cycle and the consecutive frozen-thawed embryo transfer (FET) cycles were exclusively included for this group of patients. The other group of poor responders included patients, in whom ovarian stimulation cycles exclusively involved MOS or natural cycles. Poor responders who had undergone other protocols were excluded (**Figure 1**). Additionally, patients with endometrial polyps, submucosal myomas, endometrium separation, history of multiple induced abortions (≥4 times), diagnosis of uterine adhesions, uterine malformation like Mullerian anomalies, bicornuate uterus, complete septate uterus were excluded. Patients who underwent PGT-A were also excluded. All poor responders were informed that the clinical pregnancy rate was frustratingly low, and the choices of GnRH-antagonist protocols or multiple MOS were discussed with them.

### Gonadotropin Releasing Hormone Antagonist and Minimal Ovarian Stimulation Protocols

In the flexible GnRH antagonist protocol, at least 300 IU/day recombinant follicle stimulating hormone (FSH) and/or human menopausal gonadotropin were initiated on day 2 or 3 of the menstrual period and continued daily afterward until the day of human chorionic gonadotropin (hCG) administration. The dose was adjusted according to the ovarian response. Cetrorelix (0.25 mg) was started flexibly when the follicle reached a mean diameter of 14 mm, and continued daily afterward until the day of hCG administration; hCG 6,000–10,000 IU or GnRH-agonists 0.1–0.2 mg were selectively administered for final oocyte maturation when at least two follicles reached a diameter of 17 mm.

In MOS, clomiphene at a dose of 25–100 mg was started on day 2 or 3 of the menstrual period and continued daily for 5 days, or until trigger day. Gonadotropin at a dose of 75–150 IU was selectively initiated from day 3 or 5 of the menstrual period; hCG (6,000–10,000 IU) or GnRH-agonists (0.1–0.2 mg) were selectively used as a trigger for final oocyte maturation when 1–2 follicles reached a diameter of 17 mm. In natural cycles, there is no gonadotropin or clomiphene or Letrozole administered. hCG 6000 or GnRH-agonists 0.1mg were selectively administered for final oocyte maturation. Mono-follicular development was advocated for oocyte retrieval.

Luteal phase supplementation depended on fresh embryo transfer cycle or FET cycle including artificial and natural cycle. For fresh embryo transfer cycle and FET with natural cycle, luteal phase supplementation was initiated days before embryo transfer, specifically 40 mg oral dydrogesterone per day until 12 weeks of gestation and hCG 2000 IU intramuscularly every 5 days for three times. For FET-HRT cycles, once the timing of FET was determined, administration of progesterone intramuscularly 60 mg or Crinone vaginally 90 mg was initiated daily along with 40 mg oral dydrogesterone per day and 6 mg oral estradiol per day.

### Oocyte Retrieval Laboratory Procedures

Oocyte retrieval was performed under ultrasound guidance 35–36 h after the trigger. IVF or ICSI was selectively used for fertilization. Embryos were either freshly transferred after oocyte retrieval or frozen-thawed transfer in consecutive FET cycles. All embryos were cultured in in 37°C, 5% O_2_ and 6% CO_2_ concentration. Embryo development was evaluated according to the morphological criteria. Day 2 or 3 cleavage-stage embryos with at least 3 or 6 blastomeres respectively, and less than 20% fragmentation were eligible for transfer and cryopreservation. For blastocysts, fully expanded to hatched blastocysts with inner cell mass and trophectoderm B quality (from 4BC upward) were eligible. Luteal phase supplementation was applied variably according to embryo transfer strategies and various endometrium preparation methods in FET cycles.

### Outcome Measures

The primary outcome was the CLBR per aspiration for women with a GnRH-antagonist protocol, defined as at least one delivery of a live infant resulting from one ART aspiration cycle, including fresh and FET cycles within 24 months. For women administered the MOS protocol, the CLBR per person was defined as at least one delivery of a live infant resulting from all ART cycles within 24 months (21). The number of oocytes retrieved and fertilized, number of viable embryos, financial expenditure, and time to first live birth were secondary outcomes. Cycles where no oocytes were retrieved and no viable embryos were generated were also included in this study. Women who were not followed up because of loss of contact and whose frozen embryos remained un-transferred within 24 months were considered as “not having live births”.

### Statistical Analysis

Analyses were performed according to the intention-to-treat principle. Comparisons between GnRH-antagonists and MOS were performed using the Student’s t-test, Man-Whitney U test, and chi-square tests. Student’s t-test was used where sample data were normally distributed for continuous values and the mean (± SD) was reported. Man-Whitney U test was used where sample data were not normally distributed for continuous values and the median (first quartile, third quartile) was reported. Chi-square was used for categorical values and the number was reported. We verified variables distribution by statistical tests Kolmogorov-Smirnov and Shapiro-Wilk from SPSS. Univariate regression and multivariate logistic regression were applied to identify the candidate factors predictive of CLBR. The candidate variables were the age, body mass index, basal FSH, basal estradiol (E2), infertility years, primary infertility (*vs*. secondary infertility), and ovarian stimulation protocols. All independent variables were concomitantly entered into the logistic regression model. The likelihood of CLBR was presented as an odds ratio (OR) and 95% confidence intervals (CI). All analyses were conducted using SPSS statistics. P values < 0.05 were considered statistically significant. The economic analysis included costs for pharmacological compounds and IVF procedures up to the day of pregnancy. Economic evaluation focused on direct medical costs, not including the cost of examinations before IVF treatment or any commute fees. Costs were based on Shanghai General Hospital prices and have been presented in RMB.

### Ethical Approval

Approval for this study was obtained from the institutional review board and ethics committee of the Shanghai General Hospital.

## Results

This study included 325 women (325 cycles) who underwent GnRH-antagonist ovarian stimulation and 374 patients (733 cycles) who underwent multiple minimal ovarian stimulation (MOS), including the natural cycle. Baseline demographic and clinical characteristics between the GnRH-antagonist and MOS groups were similar, although as shown in **Table 1**, the basal FSH in the MOS group was higher than that in the GnRH-antagonist group (p < 0.001). GnRH-antagonist cycles were characterized by significantly longer durations of gonadotropin (Gn) stimulation days, a higher total dose of Gn, higher peak E2, higher progesterone (P) levels, lower luteinizing hormone (LH) levels, and thicker endometrium than the MOS cycle at the trigger day (**Table 2**). GnRH-antagonists resulted in higher number of oocytes retrieved, meiosis II oocytes, fertilized oocytes, and more viable embryos than both the first MOS and cumulative stimulation of multiple MOSs (p < 0.001) (**Table 2**).

**Table 1:** Baseline demographic and clinical characteristics based on different protocols.

	GnRH-antagonist stimulation	Minimal ovarian stimulation	P
**Maternal age (year)**	38.46 ± 4.64	38.83 ± 4.75	0.328
**Body mass index**	23.57 ± 2.9	23.78 ± 3.10	0.056
**Primary infertility**	143	154	0.451
**Infertility years**	5 (2, 8)	4 (2, 7)	0.134
**Primary cause of infertility**			
**Male**	117	129	0.677
**Tubal**	209	231	0.487
**Poor ovary response**	6	25	0.002
**Endometriosis**	14	21	0.429
**Anovulatory**	10	4	0.105
**Unexplained**	12	4	0.039
**Other causes**	8	15	0.252
**Basal E2 level (pmol/L)**	145 (95.5, 211.5) (N=315)	134 (88.59, 211.00) (N=355)	0.220
**Basal FSH level (mIU/ml)**	9 (7.2, 15.1) (N=315)	11.7 (8.600, 17.725) (N=354)	<0.001

**Table 2:** Cycle characteristics according to different protocols.

	First GnRH-antagonist stimulation (325 cycles)	Minimal ovarian stimulation		P^a^	P^b^
		First (374 cycles)	Multiple (733 cycles)		
**Duration of Gn stimulation (days)**	9 (8, 10)	6 (4, 8)	/	<0.001	/
**Total dose of Gn (IU)**	2400 (1800, 2925)	900 (600.00, 1256.25)	/	<0.001	/
**Peak E2 level at trigger day (pmol/L)**	7585 (4213.5, 11666.0) (N=320)	2707 (1630, 4815) (N=365)	/	<0.001	/
**P level at trigger day (nmol/L)**	2.64 (1.623, 3.683) (N=68)	1.36 (1.032, 3.105) (N=96)	/	0.005	/
**LH level at trigger day (U/L)**	3.14 (2.205, 5.210) (N=67)	7.96 (5.318, 13.858) (N=96)	/	<0.001	/
**Endometrial thickness at trigger day (mm)**	9 (8.5, 10.4)	6 (5.0, 8.2)	/	<0.001	/
**ICSI/IVF**	100/225	104/270	/	0.390	/
**Number of oocytes retrieved**	7 (4, 10)	2 (1, 4)	4 (2, 7)	<0.001	<0.001
**Number of MII oocytes**	5 (3, 8.25)	2 (1, 3)	3 (1, 4)	<0.001	<0.001
**Number of fertilized oocytes**	5 (3, 7)	2 (1, 3)	3 (1, 5)	<0.001	<0.001
**Number of viable embryos**	2 (1, 4)	1 (0, 2)	2 (1,3)	<0.001	<0.001

a First GnRH-antagonist vs. first minimal ovarian stimulation.

b ^b^First GnRH-antagonist vs. multiple minimal ovarian stimulation.

Gn, gonatrodopin; IVF, in vitro fertilization; ICSI, intracytoplasmic sperm injection; MII, meiosis II.

As for clinical results (**Table 3**), the CLBR for both groups of patients was low. For the first IVF cycle, GnRH-antagonists demonstrated higher CLBR per aspiration than the first MOS on both, univariate analysis (12.92 *versus* 4.54%, crude OR 3.117; 95% CI 1.737, 5.592, p < 0.001) and multivariate analysis after adjusting for female age, body mass index, basal FSH, basal E2, infertility years, and primary infertility (*vs*. secondary infertility) (adjusted OR 2.606; 95% CI 1.386, 4.899, p = 0.003). Female age, basal FSH, and infertility years were independent factors negatively associated with the likelihood of CLBR per aspiration (**Supplemental Figure 1**). A cluster of multiple aspiration cycles per woman has to be considered in the MOS group. Therefore, we also measured the CLBR per person in this group of patients. The CLBR per aspiration in the GnRH-antagonist group was higher than the CLBR per person of multiple MOSs on univariate analysis (12.92 versus 7.22%, crude OR 1.907; 95% CI 1.147, 3.171, p < 0.001), while the type of ovarian stimulation (GnRH-antagonist *vs*. MOS) was not associated with CLBR on multivariate logistic regression after adjusting for the same factors (adjusted OR 1.702; 95% CI 0.971, 2.982, p = 0.063). Female age and basal FSH were the only independent factors negatively associated with the likelihood of CLBR (**Supplemental Figure 2**).

**Table 3:** Clinical outcomes according to different protocols.

	First GnRH-antagonist(325 cycles, 325 persons)(per aspiration)	First minimal ovarian stimulation(374 cycles, 374 persons)(per aspiration)	Multiple minimal ovarian stimulation(733cycles, 374 persons)(per person)	P^a^	P^b^	Adjusted OR(95%CI)^a^P^a^	Adjusted OR(95%CI)^b^P^b^
**CLBR**	42 (12.92%)	17 (4.54%)	27 (7.22%)	<0.001	0.012	2.606 (1.386, 4.899) 0.003	1.702 (0.971, 2.982) 0.063
**Cost**	20,838 (17,953, 23,422)	12,254 (9,612.5, 14,875.5)	21,261.5 (15,892.5, 35,140.25)	<0.001	0.130	/	/
**Time to First Live Birth**	9 (8, 10.75)	/	11 (9, 14)	/	0.014	/	/

a First GnRH-antagonist vs. first minimal ovarian stimulation.

b First GnRH-antagonist vs. multiple minimal ovarian stimulation.

Adjusted OR: adjusting for age, body mass index, basal FSH, basal E2, infertility years, and primary infertility (vs. secondary infertility).

CLBR, cumulative live birth rate; OR, odds ratio; CI, confidence interval.

On considering the first cycle of ovarian stimulation during economic-effectiveness analysis, the cost of using GnRH antagonists was higher than that of MOS [20,838 (17,953, 23,422) ¥ *versus* 12,254 (96,12.5, 14,875.5) ¥, p < 0.001]. However, the cumulative financial expenditure was statistically similar between one time GnRH-antagonists and multiple MOS [20,838 (17,953, 23,422) ¥ *versus* 21,261.5 (15,892.5, 35,140.25) ¥, p=0.13]. On considering the time to first live birth, GnRH-antagonists showed obviously shorter times than repeated modified natural cycles [9 (8, 10.75) months *versus* 11(9, 14) months, p = 0.014].

## Discussion

### Main Findings

In the present retrospective study on POR, patients who underwent COS with conventional GnRH-antagonist protocols had a significantly higher number of retrieved oocytes, viable embryos, and statistically similar CLBR compared to those who underwent multiple MOS; however, the time to live birth was earlier with similar financial expenditure. The GnRH-antagonist protocol is a suitable choice when developing a COS strategy plan for poor responders.

### Interpretation of Data

We evaluated whether poor responders benefit from GnRH-antagonist protocols compared to MOS, as the preferred protocol in these patients remain unclear. Although reports suggest that MOS is a relatively preferable strategy for POR, we believe that controlled ovarian hyperstimulation with daily high gonadotropin doses in the GnRH-antagonist protocol should be commonly offered to poor responders. Our observations are in accordance with research that suggests that raising FSH levels during stimulation by high-dose FSH reduces cancellation and improves clinical success (23), and mild ovarian stimulation is inferior to conventional regimen in POR in terms of retrieved cumulus oocyte complexes (22, 24). In addition, there are several studies comparing MOS and other ovarian stimulation protocols applied in POR including some RCTs (12, 15, 25–28). They suggested MOS or mild ovarian stimulation induced non-inferior successful rate with shorter duration of stimulation and economical advantages than conventional ovarian stimulation strategy (19, 29–32). The strategy of performing increasing FSH starting dose has not shown any consistent benefit. However, the main limitation of these studies is the low number of patients and the lack for data involving cryopreservation of surplus embryo and cumulative pregnancy rate. The outcomes of consecutive FET cycles are important because fresh live birth rate was negatively impacted by high dose of gonadotropin, while frozen transfer live birth rate was unaffected by total FSH dose (33). Our study is the first to evaluate the CLBR including both FET cycles and repeated MOS cycles in 2 years of follow-up.

In the context of laboratory performance, the need for the retrieval of a large number of oocytes *via* ovarian stimulation is an integral part of successful IVF treatment, since the number of oocytes and viable embryos are independent factors that increase CLBR (34). A large oocyte field is associated with an increased likelihood of CLBR per aspiration across female age. For poor responders, the pregnancy rate is reduced when fewer oocytes were retrieved. The maximum CLBR is observed when around nine oocytes are retrieved in women older than 45 years (6, 35) Any additional oocyte retrieved indicates possible improvement of CLBR for this challenging population of POR.

The higher number of euploid blastocysts correlated with a higher cumulative pregnancy rate. Reports have indicated that a higher dose of gonadotropins resulted in an increased rate of aneuploidy in embryos and granulosa cells (36). However, there are some controversies in this regard. Earlier research suggested that a higher proportion of embryos of good morphological quality are obtained with mild stimulation compared to conventional stimulation, and embryo development is adversely affected in a COS dose-dependent manner (37). However, recent studies demonstrated that aggressive stimulation does not increase the rate of embryo aneuploidy in preimplantation genetic screening (PGS) cycles in both, infertile patients and oocyte donors (38). The so-called “detrimental effect” of high dose stimulation was not evident when natural and stimulated IVF cycles were compared. The benefits of a higher number of retrieved oocytes cannot be mitigated by the age-related embryo aneuploidy rate, and can explain why high stimulation results in similar reproductive outcomes. Higher doses of gonadotropins tend to result significantly higher E2 levels on the day of hCG administration and diminished endometrial receptivity. However, the freeze-all policy and higher frequency of FET alleviates the possible negative influence of conventional high-dose stimulation on endometrial receptivity. Endometrium maybe adversely affected by high dose of gonadotropin only in fresh IVF cycle. In a retrospective analysis, Trifon et al. suggested that live births are significantly higher with modified natural cycles than with high-dose FSH stimulation GnRH-antagonists in poor responders (14). However, they only accounted for the live birth rate in fresh transfer cycles and did not consider other FET cycles, which represent the whole picture of these clinical situations.

Tilborg et al. indicated that an increased dose of FSH resulted in a statistically similar CLBR compared to the standard dose regimen, but with collateral increases in financial costs (12). Financial factors play an important role when considering the number of IVF cycles a patient will attempt, since there is no insurance coverage for IVF treatment in some countries including China. The modified natural cycle was considered to be a patient-friendly ovarian stimulation protocol. Some research has shown that multiple MOS or modified natural cycles offer a reasonable long-term success rate with less financial costs. However, a report suggested that modified natural cycles are of no benefit with a less than 1% live birth rate in genuine poor responders, who yielded up to three oocytes with conventional COS (39). The lower ongoing pregnancy rate resulting from mild stimulation was particularly related to a high cancellation rate (40). In our analysis, the total financial expenditure per person for repeated MOS was similar to that of the one-time GnRH-antagonist protocol. The drug cost linked to gonadotropin in one-time GnRH-antagonist regimen is paralleled by the clinical outcome. From our experience, in the multiple MOS strategy, the cost of repeated oocyte retrieval and embryo transfer procedures comprises most of the cumulative financial cost, while the pharmacological expenses of gonadotropin are considerably less. The whole financial expenditure of repeated MOS is not less than the conventional GnRH-antagonist regimen. Additionally, in our study, repeated MOS showed a longer time to live birth than the GnRH-antagonist protocol. Thus, repeated MOS is not as beneficial as presumed.

### Strengths and Limitations

Before the IVF treatment for these poor responders, both clinicians and patients were confronted with the high possibility of repeated stimulation cycles. In the MOS group, a cluster of multiple treatment cycles per woman has to be considered. One strength of our study was that we measured the CLBR of multiple modified natural cycles, which included not only the live birth rate from one single stimulation cycle, but also that of the consecutive cycles within 2 years of follow-up. Thus our analysis contributed important data to daily clinical practice before making the ovarian stimulation strategy for these poor responders. This research is limited by its retrospective design. Patients were allocated to two stimulation protocols based on the physician’s discretion and patient consultations; selection bias is therefore possible, and potential confounders cannot be accounted for. Poor responders are not a homogeneous group of patients, and the prognosis varies greatly depending on the age or actual number of oocytes obtained. Both, predicted and unexpected poor responders were included in our analysis. Unexpected poor responders seem to have different biological characteristics and prognosis as a different entity than the predicted poor responders (41). The heterogeneous population may have had different prognoses; this may have affected our inferences.

## Conclusions

The current study provides evidence that GnRH-antagonists are not inferior to multiple MOS in POR in terms of both, the success rate and time-and-cost-benefit analysis. While making COS strategy plans for predicted POR, this analysis may improve the counseling of IVF treatment for these poor responders and assist clinicians in determining the best candidates for the COS strategy. The GnRH-antagonist protocol enhanced the oocytes yield, did not lead to considerable cost and acted as a reasonable alternative for this difficult-to-treat group of patients.

## Data Availability Statement

The raw data supporting the conclusions of this article will be made available by the authors, without undue reservation.

## Ethics Statement

The studies involving human participants were reviewed and approved by Institutional review board and ethics committee of Shanghai General Hospital. The patients/participants provided their written informed consent to participate in this study.

## Author Contributions

YL and YW were involved in the study concept and design. YL collected the data and drafted the manuscript. RS analyzed the data. YW revised the manuscript for important intellectual content. All authors contributed to the article and approved the submitted version.

## Funding

This study was supported by a grant from the National Natural Science Foundation of China (No. 82002738). Funds were used to cover the publication fee and to appreciate the hard work of all authors.

## Conflict of Interest

The authors declare that the research was conducted in the absence of any commercial or financial relationships that could be construed as a potential conflict of interest.

## Supplementary Material

Supplementary Figure 1 The odds of achieving CLBR per aspiration between first GnRH antagonist administration and first MOS.

Supplementary Figure 2 The odds of achieving CLBR per person between first GnRH antagonist administration and multiple MOS.

## Abbreviations

CLBR, cumulative live birth rate; FET, frozen-thawed transfer; MOS, minimal ovarian stimulation; GnRH-antagonist, gonadotropin releasing hormone antagonist; ART, assisted reproductive technology; COS, controlled ovarian stimulation; POR, poor ovary responder; ESHRE, European Society of Human Reproduction and Embryology; GH, growth factor; DHEA, dehydro-epiandrosterone; LPS, luteal-phase stimulation; FPS, follicular-phase stimulation; ITT, intention to treat; IVF, *in vitro* fertilization; 95% CI, 95% confidence interval; PGS, preimplantational genetic screening; FET, frozen-thawed embryo transfer.
