# Supplementary figures and images for: Cumulative Live Birth Rate and Cost-Effectiveness Analysis of Gonadotropin Releasing Hormone-Antagonist Protocol and Multiple Minimal Ovarian Stimulation in Poor Responders
Source: Front Endocrinol (Lausanne). 2021 Jan 14;11:605939. doi: 10.3389/fendo.2020.605939 (PMC7841408; doi:10.3389/fendo.2020.605939)

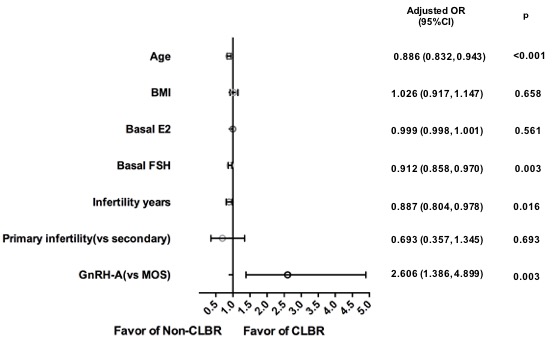

Supplement: Supplementary Figure 1 — The odds of achieving CLBR per aspiration between first GnRH antagonist administration and first MOS. [file Image_1.jpeg]

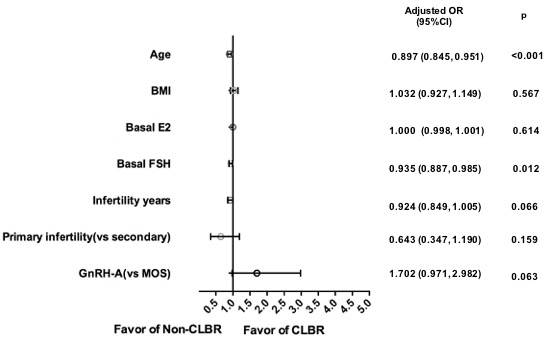

Supplement: Supplementary Figure 2 — The odds of achieving CLBR per person between first GnRH antagonist administration and multiple MOS. [file Image_2.jpeg]
